# Geospatial resources for supporting data standards, guidance and best practice in health informatics

**DOI:** 10.1186/1756-0500-4-19

**Published:** 2011-01-26

**Authors:** Tony Mathys, Maged N Kamel Boulos

**Affiliations:** 1 EDINA National Data Centre, The University of Edinburgh, Edinburgh, UK; 2 Faculty of Health, The University of Plymouth, Drake Circus, Plymouth, Devon, PL4 8AA, UK

## Abstract

**Background:**

The 1980s marked the occasion when Geographical Information System (GIS) technology was broadly introduced into the geo-spatial community through the establishment of a strong GIS industry. This technology quickly disseminated across many countries, and has now become established as an important research, planning and commercial tool for a wider community that includes organisations in the public and private health sectors.

The broad acceptance of GIS technology and the nature of its functionality have meant that numerous datasets have been created over the past three decades. Most of these datasets have been created independently, and without any structured documentation systems in place. However, search and retrieval systems can only work if there is a mechanism for datasets existence to be discovered and this is where proper metadata creation and management can greatly help.

This situation must be addressed through support mechanisms such as Web-based portal technologies, metadata editor tools, automation, metadata standards and guidelines and collaborative efforts with relevant individuals and organisations. Engagement with data developers or administrators should also include a strategy of identifying the benefits associated with metadata creation and publication.

**Findings:**

The establishment of numerous Spatial Data Infrastructures (SDIs), and other Internet resources, is a testament to the recognition of the importance of supporting good data management and sharing practices across the geographic information community. These resources extend to health informatics in support of research, public services and teaching and learning.

This paper identifies many of these resources available to the UK academic health informatics community. It also reveals the reluctance of many spatial data creators across the wider UK academic community to use these resources to create and publish metadata, or deposit their data in repositories for sharing.

The Go-Geo! service is introduced as an SDI developed to provide UK academia with the necessary resources to address the concerns surrounding metadata creation and data sharing. The Go-Geo! portal, Geodoc metadata editor tool, ShareGeo spatial data repository, and a range of other support resources, are described in detail.

**Conclusions:**

This paper describes a variety of resources available for the health research and public health sector to use for managing and sharing their data. The Go-Geo! service is one resource which offers an SDI for the eclectic range of disciplines using GIS in UK academia, including health informatics.

The benefits of data management and sharing are immense, and in these times of cost restraints, these resources can be seen as solutions to find cost savings which can be reinvested in more research.

## Background

The 1980s marked the occasion when GIS technology was initially introduced into the geospatial community. (The 1980s mark the establishment of a strong GIS industry and new vendor products, with major influence on the discipline of Geography, though earlier non-mainstream systems also existed, e.g., Canada Geographic Information System in the mid-1960s [1].) This technology quickly disseminated across many countries, and has now become established as an important research, planning and commercial tool for a wider community that includes organisations in the public and private health sectors.

The broad acceptance of GIS technology, and the nature of its functionality, has meant that numerous datasets have been created over the past three decades. Most of these datasets have been created independently, and without any structured documentation systems in place. A 2006 spatial data audit conducted at four UK academic institutions is a testament to this. The audit yielded more than 500 spatial dataset titles, and also found hundreds more files in personal computer directories and in stored media [2,3]. These files had no provenance or descriptive documentation, but their extensions revealed that they had been created with various GIS and remote sensing software packages.

Eschewing metadata creation and publication is pervasive across the (Geographic Information) GI-community; the reasons being that it is perceived to be tedious and time-consuming [4,5]. A survey of archaeological organisations in the Republic of Ireland revealed that 57.1 percent of these organisations did not include metadata creation as part of their organisations' data management strategy [6]. The Open Geospatial Consortium (OGC) Working Group on Data Quality conducted a survey in 2008 which revealed that about 55.8 percent of respondents said they were not using any recognised standards for data quality work being conducted in their organisation [7]. Academia appears to represent a greater challenge based on the lack of commitment to metadata creation despite support and engagement with that community in the UK [8].

Undocumented spatial datasets face a number of risks. Undocumented dataset files are likely to become redundant. If familiarity with a dataset is lost then time and costs must be assumed for any reassessments. The absence of a formal documentation system also means that datasets cannot easily be revealed to the wider geo-spatial community, therefore limiting other potential users' ability to locate both the datasets and developers. The absence of information about existing datasets can lead other organisations to expend considerable time and costs in producing data that are already in existence, but stored at an undisclosed location.

This widespread lack of proper data documentation can be improved through support mechanisms such as geospatial metadata standards and guidelines, training, metadata editor tools and metadata automation to extract information from spatial datasets. Web-based resources such as geoportal technologies must also be developed to publish metadata. A geoportal is a type of Web portal used to find and access geographic (geospatial) information and associated geographic services (display, editing, analysis, etc.) via the Internet [9]. Geoportals are important for effective use of GIS and are key to the support of Spatial Data Infrastructures (SDIs) [10].

Successful delivery of metadata and spatial datasets via geoportals and repositories can result in the establishment of SDIs across the various GI-community sectors [11]. These can provide the infrastructure to support good data management practices, facilitate the exchange of information to advance research and teaching in academia, and to aid planners and policy makers in the public sector.

WMS (Web Map Service), WFS (Web Feature Service), and WCS (Web Coverage Service) are three Web service standards from the Open Geospatial Consortium (OGC) which also provide critical SDI support. These allow Web clients to query and receive geographic information in the form of image, vector, or coverage data. The open source GeoServer application is the reference implementation of a server for the WMS, WFS, and WCS standards [12].

It is also important to engage with data developers to implement a policy of data management and sharing using these resources. The challenge lies with encouraging and sustaining these activities through identifying the benefits associated with metadata creation and publication and data sharing.

Ultimately, the driving force for implementation rests in the hands of individuals and organisations. Motivating people to create metadata remains the greatest challenge and requires imagination and guidance through a range of initiatives promoting metadata creation and publication in the context of the aforementioned standards, support mechanisms and identified benefits.

### On metadata and metadata standards

The US Federal Geographic Data Committee (FGDC) describes geospatial metadata as follows: "*A metadata record is a file of information, usually presented as an XML (eXtensible Markup Language) document, which captures the basic characteristics of a data or information resource. It represents the who, what, when, where, why and how of the resource. Geospatial metadata are used to document geographic digital resources such as Geographic Information System (GIS) files, geospatial databases, and earth imagery. A geospatial metadata record includes core library catalogue elements such as Title, Abstract, and Publication Data; geographic elements such as Geographic Extent and Projection Information; and database elements such as Attribute Label Definitions and Attribute Domain Values*." [13]

Growing attention to the critical importance of geospatial metadata throughout the past three decades sparked the development of a range of metadata collection initiatives that followed various formats within different communities of practice, agencies, and countries. The task of harmonising the plethora of overlapping formal and de facto metadata standards was initially undertaken by the FGDC with the release of the Content Standard for Digital Geospatial Metadata (CSDGM) in 1994 [14].

The FGDC CSDGM standard was implemented across the international GI-community, but from 1999 to 2002, the ISO/TC 211 [15] worked towards publishing a new standard which culminated in the release of ISO 19115 'Geographic information - Metadata' standard in 2003 [16]. Individual countries, groups and communities of practice are in the process of rewriting their previously-used metadata standards as "profiles" of ISO 19115, sometimes with the inclusion of additional metadata elements as extensions to the ISO standard, e.g., the North American Profile (NAP) [17]. ISO 19139 [18] provides the XML implementation schema for ISO 19115, specifying the metadata record format to describe, validate, and exchange geospatial metadata in XML.

### Implementation

The introduction of these geospatial metadata standards also required initiatives to encourage their uptake. In the late 1990s, the FGDC took the lead as part of an effort to implement a National Spatial Data Infrastructure (NSDI) for the United States and other countries [19]. The FGDC provided seed money to geospatial organisations across the US to support the development of metadata and related services and geoportals at the federal, state and local levels.

This funding also targeted a range of outreach activities in support of metadata creation. Metadata workshops, presentations and academic curriculum development have been conducted over the years. The FGDC has also published numerous documents and publicity materials [20] in support of metadata creation and producing quality records, and metadata creation and editing tools, software and utilities [21]. Business cases for the benefits of metadata creation have been provided as well to encourage uptake. Interested readers may refer to the FGDC's 'The Business Case for Metadata' section in [13].

The ratification of ISO 19115 in 2003 provided the impetus for metadata initiatives around the world. Across the Atlantic, continental Europe and the UK embraced the INSPIRE Directive [22]. In September 2001, representatives from the European Commission, the European Environment Agency (EEA) and nominated representatives of the Member States' environmental and geographic information communities convened in Brussels, Belgium to set out a legislative framework which would subsequently aim at creating a European Spatial Data Infrastructure (SDI). This SDI would be based on Member States' infrastructures, and intended to improve interoperability and delivery of environmental information across all Member States of the EU. Each Member State would be required to provide metadata catalogues to allow users to identify available data and geoservices information, and deliver online data discovery, viewing, downloading, and transformation services. The INSPIRE Directive came into force on 15 May 2007 and is being implemented in various stages, with the year 2019 targeted for full implementation [23].

## Health Spatial Data Infrastructures (SDIs)

Health informatics organisations have established a range of portals and repositories to deliver metadata, spatial data and interactive mapping features to medical researchers, public health officials and the general public.

These online resources provide environmental and demographic data which are of particular benefit to epidemiology researchers. Standards, metadata and good data management practices become essential in the delivery of this information to this range of users [24-26]. Also of particular importance is the promotion of metadata creation through changing the perceptions of metadata in public health culture among public health professionals [26]. It is worth noting in this respect that ISO 19115 Topic Categories include one dedicated to health (health services, human ecology, and safety; for example, resources describing human disease and illness, factors affecting health, hygiene, mental and physical health, substance abuse, and health services) [16].

The following lists provide examples of health-related portals which deliver important information and data via interactive mapping, metadata and download services. Interactive mapping is often supported with Web Map Service (WMS), Web Feature Service (WFS), and Web Coverage Service (WCS).

### Examples of data and interactive mapping portals for medical researchers and public health

**Figure 1:**
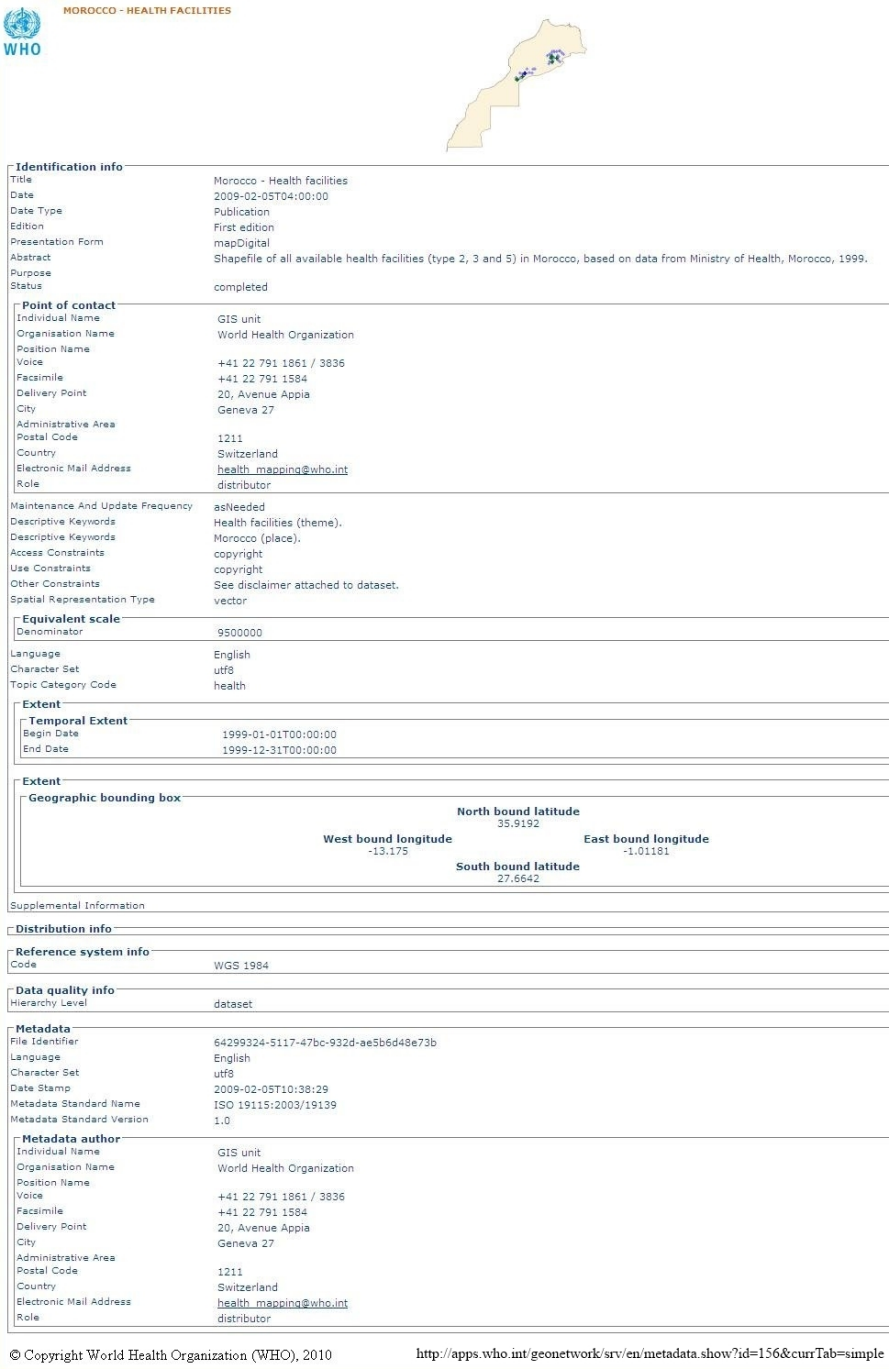
**A WHO GeoNetwork metadata record example**. WHO GeoNetwork metadata record (human-readable form) for 'Morocco - Health facilities'.

**Figure 2:**
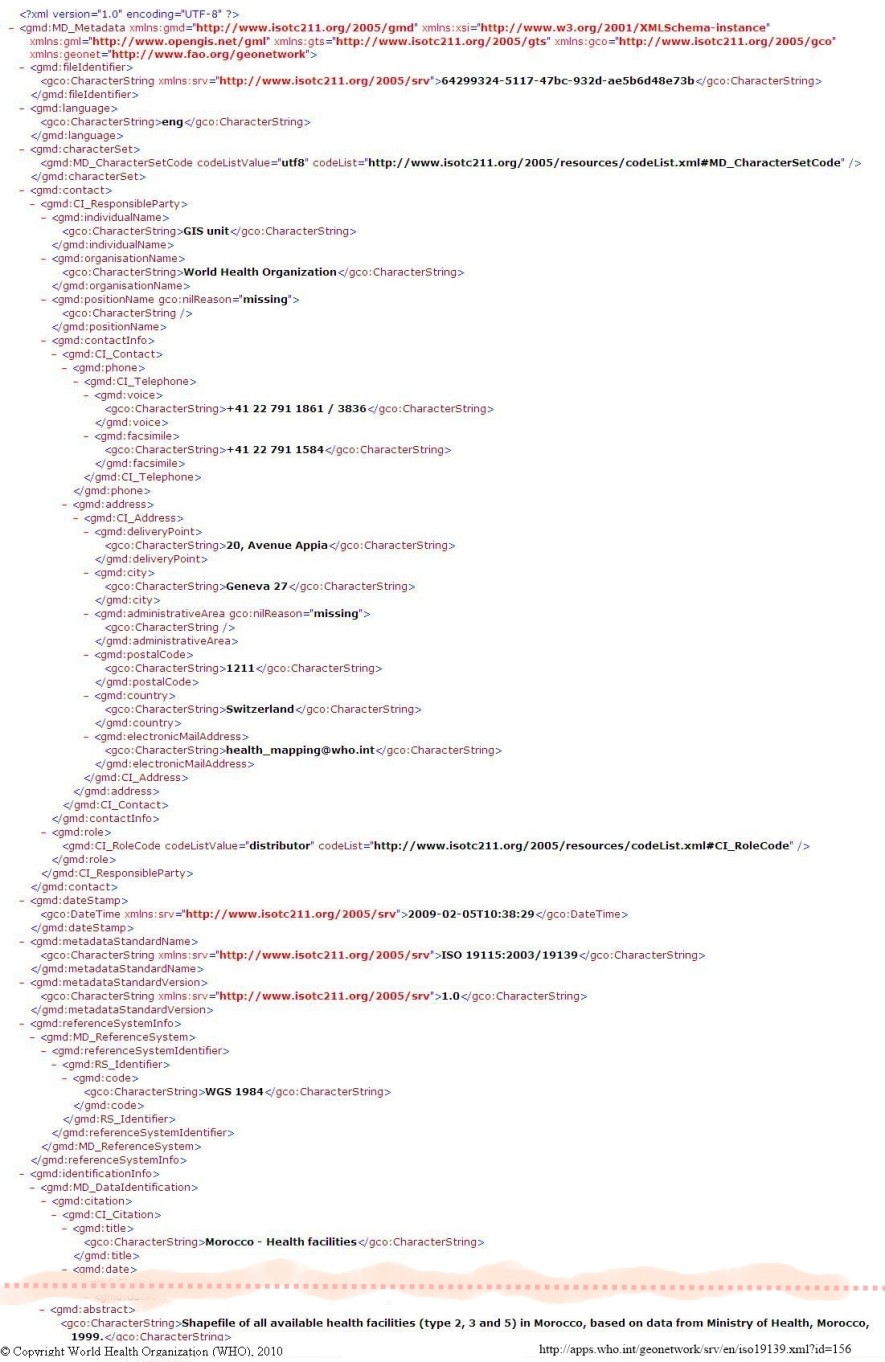
**A WHO GeoNetwork ISO 19139 XML metadata record example**. Part of the WHO GeoNetwork metadata record (ISO 19139 XML version) for 'Morocco - Health facilities'.

The World Health Organization (WHO) delivers interactive maps, spatial data, satellite imagery and related applications through a portal [27] built with GeoNetwork opensource [28], a catalogue application to manage spatially referenced resources. It provides powerful metadata editing and search functions as well as an embedded interactive Web map viewer. The WHO Portal delivers metadata using the ISO 19115 Geographic Information Metadata Standard [16] (Figures 1 and 2). GeoNetwork opensource is becoming the primary source for most in the GI-community to use as an ISO 19115-compliant catalogue service for portals. The WHO also supports the Pan American Health Organisation's (PAHO) portal [29], which delivers health data and statistics and interactive maps for South American countries.

HIVmapper [30] and the HIV Spatial Data Repository [31] provide online maps and geographically-linked HIV-related data for GIS mapping. The services are supported by USAID and the US President's Emergency Plan for AIDS Relief (PEPFAR), the US Government initiative to support partner nations around the world in responding to HIV/AIDS. An example metadata record for Armenia from the HIV Spatial Data Repository can be found at [32].

The Earth Space Agency (ESA) and the UN Food and Agriculture Organization (FAO) have provided a joint contribution to the GEOportal [33], which provides an entry point to access Global Earth Observation System of Systems (GEOSS) information and services. It connects to a system of existing portals, addressing the GEO Societal Benefit Areas globally while also providing national and regional information to enhance understanding. GEOportal also provides the functionality to harvest metadata from portals built with GeoNetwork opensource. It offers a comprehensive series of health-related datasets which support prevention, early warning, research, health-care planning and delivery, and public alerts.

The Emergency and Disaster Information Service - EDIS [34] offers AlertMap, a map which provides global coverage and displays locations where epidemics, biological hazards and other natural and catastrophic events are taking place.

The Montreal Epidemiological and Geographical Analysis of Population Health Outcomes and Neighbourhood Effects (MEGAPHONE) catalogue [35] is a spatial data infrastructure developed at the Centre de Recherche du Centre Hospitalier de l'Université de Montréal to support research for documenting, analysing and understanding environmental influences on population health. Built with GeoNetwork opensource, MEGAPHONE provides an extensive spectrum of spatial databases enabling the characterisation of places and their contextual and compositional attributes in the Montreal region, Canada. Contextual and compositional indicators can be analysed alone or in relation to linked health outcomes data. By enabling comprehensive characterisations and analyses of exposures to physical, social and built environmental factors, MEGAPHONE contributes to advancing understanding of how the environment affects health. MEGAPHONE is currently used in a broad variety of population and public health research projects on topics including obesity, healthful aging, transmission of HIV and HCV, smoking habits, mental health, cardiometabolic diseases, adverse birth outcomes, and mortality.

The National Cancer Institute in the U.S. offers an interactive map for the Breast Cancer Relative Incidence Long Island [36]. The map shows variations in the ratios of observed to expected incidence of breast cancer by ZIP Code over a 5-year period in Long Island, New York.

OneGeology Portal [37] is an example of a portal which can deliver environmental data for medical research. OneGeology is intended to provide global coverage and includes geological unit and lithological data for some countries, which can be used for radon studies. The portal is built with the latest computing technology using Geoscience Markup Language (GeoSciML) [38], a GML Application Schema that can be used to transfer information about geology, with an emphasis on the "interpreted geology" that is conventionally portrayed on geologic maps.

### Interactive mapping portals for the public

The following interactive mapping portals offer a sample of information services for the public to assess health-related threats. Some of these allow users to enter postcode, place name or address details to retrieve information and map displays specific to their locations.

The European Environment Agency's Eye on Earth portal [39] provides users with information about air quality which is collected from 1,000 air quality monitoring stations across Europe. The portal also delivers water quality information at 22,000 bathing sites across Europe.

The Flood Maps for England and Wales [40] and Indicative River and Coastal Flood Map for Scotland [41] provide the public with flood risk information via interactive maps. Postcode, town, river and place name values can be entered to generate local maps displaying areas prone to flooding.

The Noise Mapping for England [42] and Scottish Noise Mapping [43] interactive maps display of industrial site locations and transport (roads, airports, rail) networks and estimated noise level bands based on modelling traffic volume.

The 3-D Map of Air Pollution for London [44] interactive map provides current air pollution levels for users. Postcodes can be entered or London boroughs selected from a drop-down list.

## The Go-Geo! SDI Service

### Go-Geo! Portal

**Figure 3:**
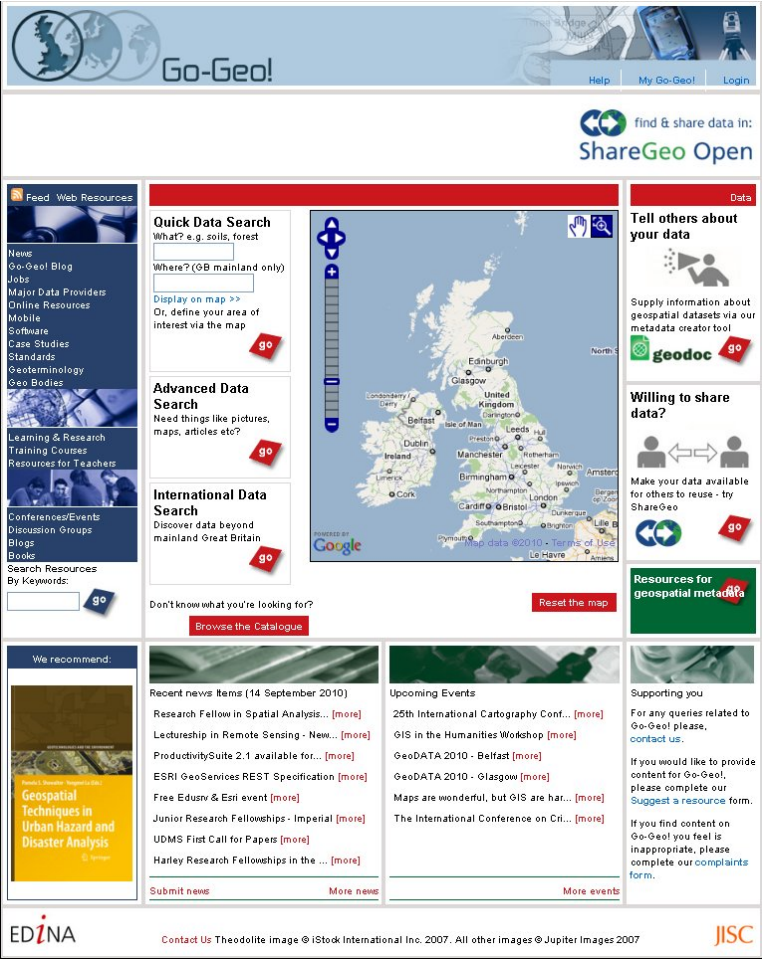
**Go-Geo! Portal**. Home page of the Go-Geo! Portal.

Go-Geo! [45] (Figure 3) is a Joint Information Services Committee (JISC) [46] funded UK academic spatial data portal service which is operated and maintained at EDINA [47], a JISC national data centre based at The University of Edinburgh. Go-Geo! serves as an online resource discovery tool which allows for the identification and retrieval of records describing the content, quality, condition and other characteristics of spatial data which exist within UK tertiary education and beyond. The portal offers interactive maps, grid co-ordinates and place names, as well as the more traditional topic or keyword to support users with spatial searching. Go-Geo! delivers an SDI to UK academia providing an online metadata editor tool (Geodoc), spatial data repository (ShareGeo), and a range of support resources including eLearning objects, metadata workshops and metadata quality assurance reviews.

The Go-Geo! portal started as a project in 2001 and became a fully funded service in 2008. This project commenced as a three year collaborative effort between EDINA and the UK Data Archive (UKDA) [48], at the University of Essex, with the JISC Portals Programme [49] providing the funding. Primary efforts were directed at the technical development of the Go-Geo! portal, and the collection of geographical information and metadata for content. Subsequently, Go-Geo! was enhanced and redesigned to improve functionality, appearance and ease of use. Other resources were added as well for facilitating metadata creation, supporting teaching and learning in the geo-related disciplines and publishing metadata for international spatial data.

A key feature of Go-Geo! is that it allows users to find related resources, such as books, photographs, projects and maps, for their geographic area of interest. Most notable is the COPAC [50], the National, Academic, and Specialist Library Catalogue, which holds 32 million records from merged UK and Irish and national libraries. Go-Geo! also allows users to search for images from the British Geological Survey JIDI Photographic Collection [51]. These and other resources are discovered by cross searching a range of online information services within and beyond the JISC Information Environment [52]; therefore, the focus of the portal is on where a resource relates to and less on what it is about, which is the focus of other JISC -funded portals.

**Figure 4:**
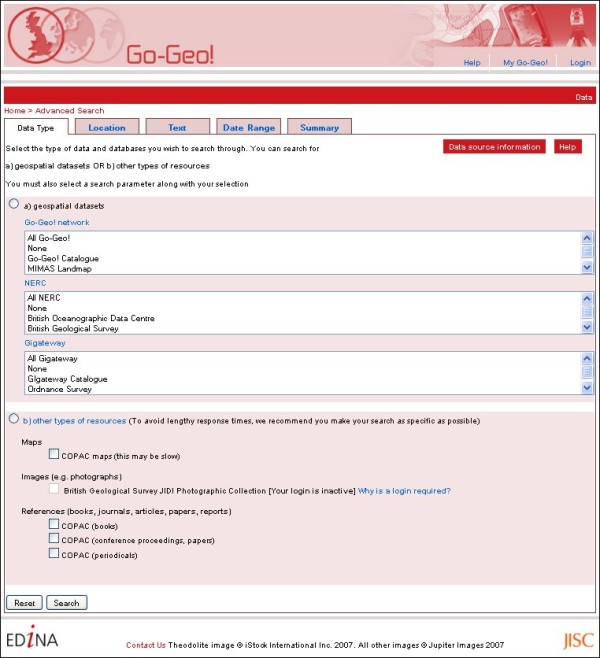
**Unlock place name search for metadata**. The Go-Geo! portal's 'Advanced Search' page showing the range of geographic resources and catalogues which can searched across the UK network.

The Go-Geo! portal also allows users to simultaneously search across UK metadata catalogues (Figure 4) including the

• National Environment Research Council's (NERC) Data Centre catalogues [53]

• Arts and Humanities Data Services (AHDS) [54]

• National Soils Research Institute [55] and

• UK Data Archive's catalogue collections [56].

The current national, government funded GIgateway portal [57] can also be searched using the Go-Geo! portal. Reciprocally, Go-Geo! serves as the Virtual Academic Catalogue (GVAC) node for GIgateway; however, work is afoot to replace this with a metadata catalogue using UK GEMIN 2.1, an INSPIRE-compliant metadata standard for the GI-community in the UK [58]. The INSPIRE Metadata Implementing Rules are guidelines with elements derived from ISO 19115 [59]. The Go-Geo! catalogue currently holds 657 metadata records with hundreds more harvested from other UK catalogues and delivered to Go-Geo! portal users.

Another important feature for users of Go-Geo! is the geographic information resource section, which provides comprehensive information about training courses, learning materials, news, events, jobs, geographical information organisations, books and journals, online services, software, standards, data providers and more. GI-related resources are added to Go-Geo! on a daily basis with almost 3,000 added to date.

**Figure 5:**
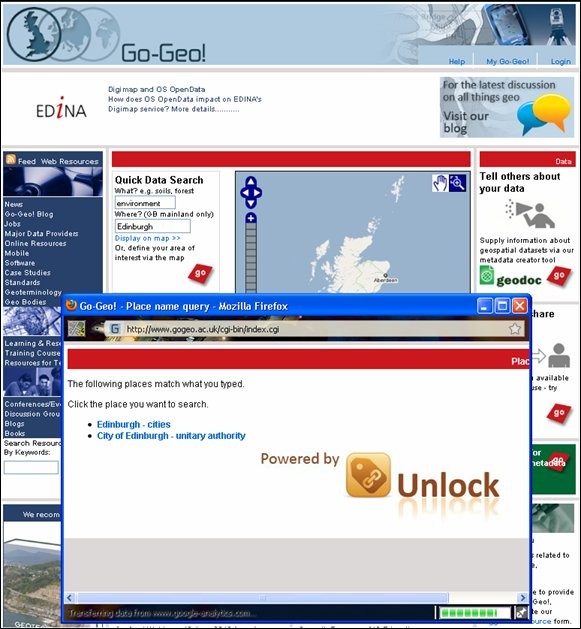
**Unlock place name search for metadata**. Searching the Go-Geo! portal's metadata catalogue using the Unlock middleware. service.

Go-Geo! also includes support for place name clarification. Unlock [60], originally known as geoXwalk, is a JISC-funded middleware service which was developed in part to support geographic searching of Go-Geo! (Figure 5). Unlock can translate a place name term into a different geography that the searched target can understand. This 'crosswalk' capability of crossing different geographies to return geographical 'equivalents' significantly improves the utility of the original search target.

### UK Academic Geospatial Metadata Application Profile

The UK Academic Geospatial Metadata Application Profile (UK AGMAP 2) is a template designed to comply with INSPIRE and support the documentation and discovery of spatial data, data series and geo-services within UK academia. UK AGMAP 2 contains all the elements required to generate compliant metadata records for the Go-Geo! portal. The UK AGMAP 2 profile was created to achieve the following goals:

• serve as a metadata element field template for the Go-Geo! portal and metadata editor tool;

• deliver support of spatial data and services documentation across an eclectic academic community, which includes extending it to support specific requirements of any discipline;

• facilitate data management and sharing requirements within academia;

• ensure compliance with the ISO 19115, INSPIRE and UK GEMINI 2.1, a UK standard which was ratified in 2010 and is compliant to INSPIRE; and

• provide the academic user with the information necessary to assess the data, and the necessary contact details to enquire further about access and use.

A guidelines document of almost 200 pages was written to serve as a reference for users of UK AGMAP 2. Use of the guidelines can also ensure that compliant metadata records are created for publication on the Go-Geo! portal site or for local data management schemes. The guidelines also represent a critical resource for improving metadata quality and currency. The guidelines also include examples to provide greater clarity for the user. Many examples are presented in a manner which can be understood across social and physical science.

Metadata records displayed on the Go-Geo! portal include direct links from the individual elements within the record to the corresponding information in the guidelines. A user unfamiliar with a metadata element displayed in a record can click it and information for that element is delivered in a new window.

The UK AGMAP 2 profile has also been cross-mapped to other relevant standards. These mappings were done to assess compatibility, and address interoperability and harmonisation among standards to improve cross-searching capabilities. Cross-mapping was also done to support the export of XML metadata records into the following formats using the Geodoc metadata editor tool:

• ISO 19115 Geographic Information Metadata Standard;

• Federal Geographic Data Committee's (FGDC) Content Standard for Digital Geospatial Metadata (CSDGM);

• UK GEMINI 2.1;

• INSPIRE;

• Dublin Core; and

• Data Documentation Initiative (DDI) Standard.

Dublin Core (ISO 15836) represents a set of 15 metadata elements which can be used to provide basic descriptive information (title, abstract, date, identifier) about a resource for cataloguing purposes [61]. The descriptive information for most spatial data is too complex for Dublin Core, hence the publication of the FGDC Content Standard for Digital Geospatial Metadata in the middle 1990s in the US, with uptake across the international GI-community. Subsequently, in 2003, the ISO 19115 standard was ratified as a machine-friendly version of the FGDC standard. The ISO 19115 standard is intended to facilitate the search and harvest of metadata records from all portal catalogues. The ISO 19115 standard also allows for GI-related groups to create profiles or guidelines for their respective communities; this is the case with INSPIRE for the European GI community, and UK GEMINI 2, which is INSPIRE-compliant and serves the GI community in the UK.

As noted, the UK AGMAP 2 Profile comprises these elements to be compliant with ISO 19115, INSPIRE and UK GEMINI 2. It is also compliant with the Data Documentation Initiative (DDI) Standard. The Data Documentation Initiative (DDI) is an effort to create an international standard for describing social science data, most of which have a spatial context [62]. The assumption is that about 80 percent of all data have a spatial context including health care data [63].

### Geodoc Metadata Editor Tool

**Figure 6:**
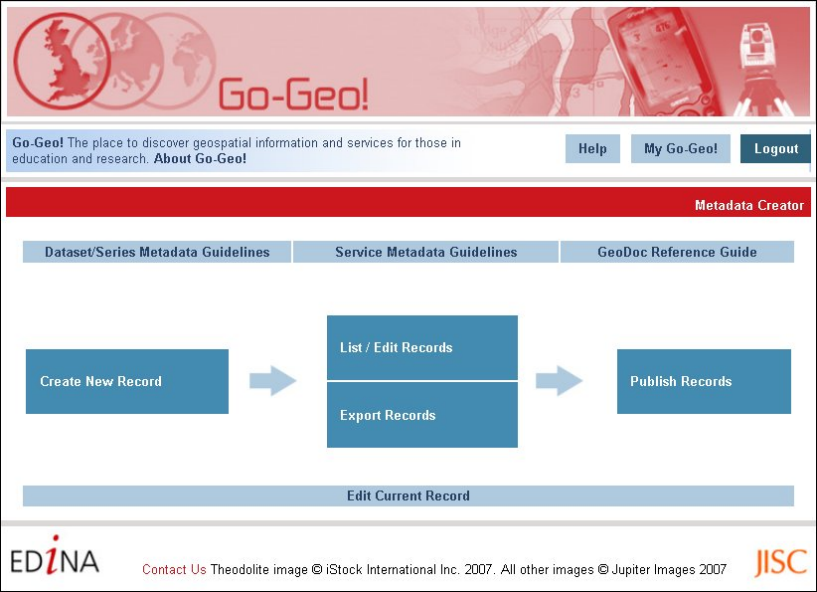
**Geodoc Metadata Editor Tool's home page**. The Geodoc Metadata Editor Tool's home page.

**Figure 7:**
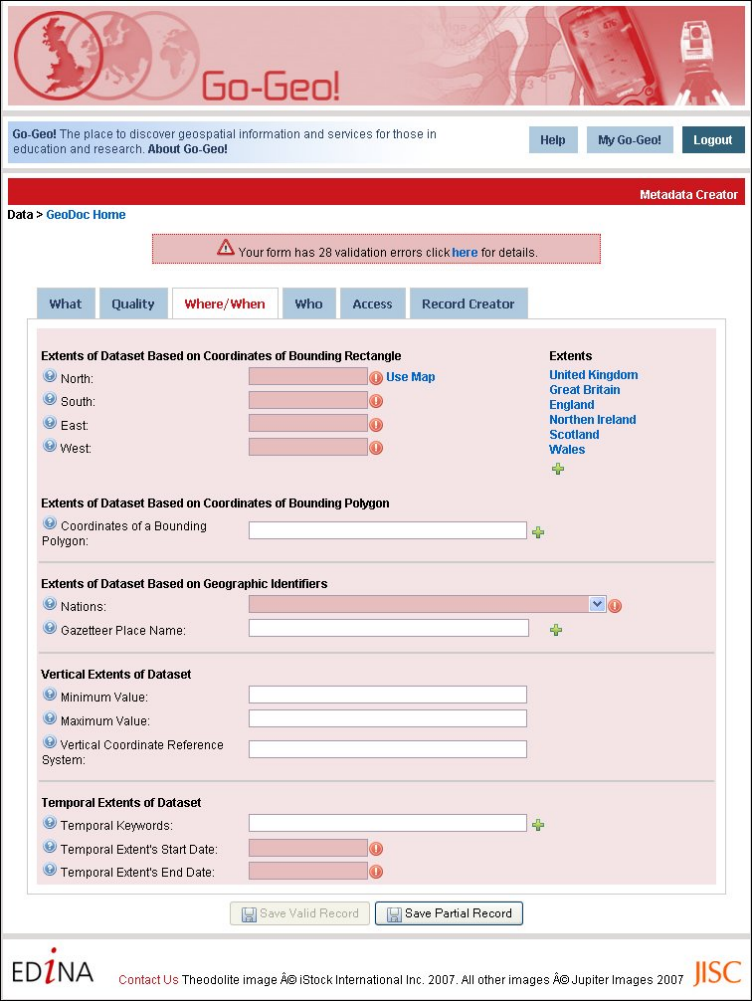
**Geodoc Metadata Editor Tool form template**. The 'Where/When' Table of Geodoc's form template.

Geodoc (Figure 6 and Figure 7) is an online Java-built metadata editor tool which delivers the UK AGMAP 2 profile to the academic community for creating discovery and descriptive level geospatial metadata records and geo services. Geodoc is accessible via the Go-Geo! portal home page [45] through an authentication service available to only those affiliated with UK academia. This restricted access allows users to create metadata with Geodoc and store records in a local and private directory, which no one else can access. This is intended to encourage metadata creation amongst those with concerns over Intellectual Property Rights (IPR), or sensitive data, but wanting to document their datasets for data management purposes. The Go-Geo! service offers support for the creation of institutional nodes, which reveal metadata to only those affiliated with an institution. This functionality can be extended to allow peers within a research group, but different academic affiliations, to create and share metadata amongst themselves for the purpose of project data management. The Go-Geo! portal provides the interface for searching their metadata, but only through the authentication log-in service, which reveals the private node created for this group or institution.

Geodoc offers a range of tools in support of metadata creation, management, publication and data sharing. In terms of functionality, Geodoc delivers a map interface to capture bounding box extent coordinates, with pre-defined bounding box extents to select for the UK, Great Britain, England, Northern Ireland, Scotland and Wales; the automation of contact detail information which populates relevant fields for each new metadata record; metadata record validation; edit directory function for storing metadata records; XML export function to aforementioned standards including INSPIRE and UK GEMINI 2; direct links between Geodoc elements and supporting text in UK AGMAP 2 guidelines; and a publication function which allows user to submit records for publication on Go-Geo! or a private/institutional node.

A geospatial metadata officer reviews all submitted Geodoc metadata records to ensure they are compliant and meet quality standards for publication on Go-Geo! or an academic institutional node. The Go-Geo! service offers academic institutions the option of publishing metadata records on private nodes. This allows only those affiliated with the institution to access the metadata in their node's catalogue via an online authentication service. The creation of institutional nodes is intended to address any concerns that might arise over Intellectual Property Rights (IPR). Data developers are also encouraged to submit their datasets to the geospatial metadata officer for extracting information to create partial metadata records which can be returned to a dataset submitter for completion.

Geodoc can also serve as a teaching and learning resource in the UK academic community. Academics can use Geodoc and the Go-Geo! portal to introduce metadata to students. This is critical in changing the mindset for prioritising data management and sharing amongst future GI professionals and academics in the UK.

### ShareGeo Spatial Data Repository

**Figure 8:**
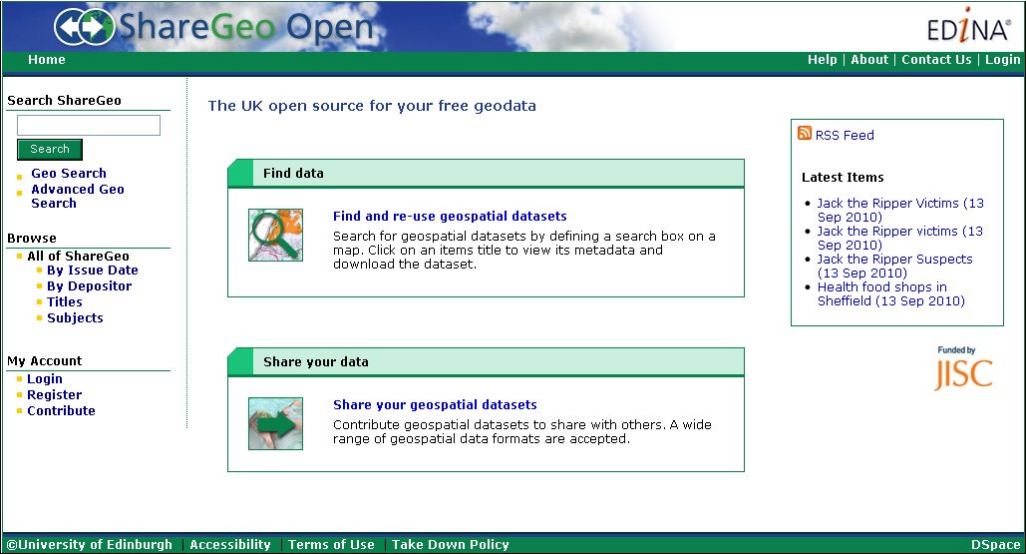
**ShareGeo Open**. Home page of ShareGeo Open Spatial Data Repository.

**Figure 9:**
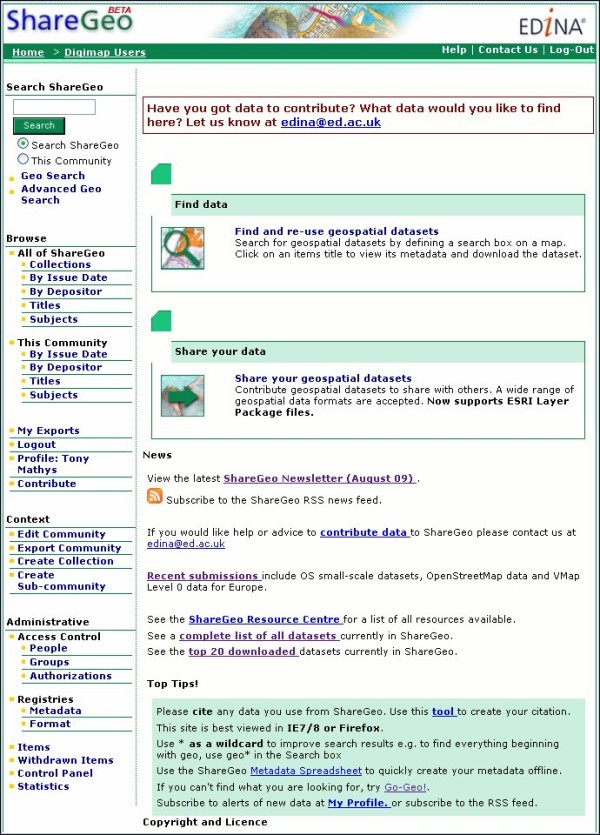
**Closed version of ShareGeo**. Home page of the closed version of the ShareGeo Open Spatial Data Repository.

Another important component to the Go-Geo! SDI is the ShareGeo spatial data repository which supports data sharing between creators and users of spatial data. Two versions, built using DSpace [64] exist for UK academic researchers, students and lecturers to deposit and download data. ShareGeo Open [65] (Figure 8) accepts and holds spatial data with no licensing or Intellectual Property Rights (IPR) restrictions; a closed version of ShareGeo (Figure 9) resides within the Digimap Collection services [66] and can only be accessed through an authentication service. This version is intended to hold spatial data derived from licensed data made available through the Digimap Collections, which include data products from the Ordnance Survey (OS) and others. ShareGeo Open was launched September 2010 and holds 95 datasets of various themes. The closed version of ShareGeo in Digimap Collections contains 101 datasets.

The spatial data landscape is changing with the UK Government calling for more OS data to be made free to the public and to stimulate growth in the GI industry (Table 1). The availability of OS OpenData products [67] to data users should also provide the impetus for more data creation, hence more content for the ShareGeo Open repository.

**Table 1:** Table showing which OS OpenData products released to the public

1:250,000 Scale Colour Raster
MiniScale

Meridian

Strategi

Land-Form Panorama Contour

Land-Form Panorama DTM

Boundary-Line

Code-Point Open (point data only; subset of Code-Point

1:50,000 Scale Gazetteer

OS Streetview

OS Locator

OS VectorMap District

### Other resources

Metadata Workshops are offered as part of an effort to change the mindset and culture of the academic community. The workshops provide an overview of metadata, standards, portals, and stress the importance of creating metadata records for supporting data sharing and good data management practices. Workshop participants are also informed about the benefits associated with creating and publishing metadata on a geoportal, including protection of investments of time and cost dedicated to data development; maintenance of an inventory of datasets to reduce time required to reassess existing datasets for new and future applications; ensuring integrity of existing and new datasets using metadata as a tracking mechanism to monitor changes and edits to datasets (version control); minimisation of the disruptive effects of staff turnover; elimination or reduction of the risk of redundancy in data collection or deletion of existing datasets; compliance with the EC INSPIRE Directive that requires metadata creation at the public sector level including academia [14,15]; and how metadata publication can greatly facilitates data discoverability, which in turn encourages collaboration.

Workshop participants learn how a geoportal can serve as a repository to store and manage metadata, thus savings in cost and time; how to use metadata to announce data and applications; how to advertise (and, where applicable, sell) spatial datasets to other interested parties; how published metadata can be referenced and cited for project proposals; and how metadata can be configured as an internal resource to access and share datasets.

The INSPIRE Directive is also introduced at workshops with an explanation about its implications for the UK academic community and those students taking employment in the public sector. Moreover, the workshops offer participants the opportunity to conduct hands-on exercises for the Go-Geo! portal and Geodoc metadata editor tool to develop a familiarity with their functionality and recognise and appreciate the simplicity involved in the creation and publication of metadata records. There have been 32 workshops run over five of the past seven years at 24 UK academic institutions with an average of 14 people attending each workshop.

The Go-Geo! portal also provides eLearning objects (Figure 8) in the form of animations and interactive modules about metadata and geospatial metadata; the latter provides a scenario which shows the steps of capturing and processing data to create a spatial dataset. This serves as a bridge for transferring this information to a metadata record using the Geodoc metadata editor tool. The geospatial metadata module introduces the user to more complex information of a dataset including positional accuracy, lineage, completeness and scale.

A biannual metadata newsletter is also published and disseminated to almost 200 lecturers and researchers across UK academia. Go-Geo! Metadata News provides readers with updates on Go-Geo! services and news about events and activities across the GI-community which would be of interest to the readers.

## Discussion and conclusions

### Challenges

Good data management and sharing practices are vital to the efficient use of geospatial information. The establishment of numerous spatial information infrastructures is a testament to the recognition of this fact, but there are still significant challenges to ensure these investments are not wasted.

Technological advances and software engineering, standards and guidance are important instruments for the development of SDIs; however, reciprocal effort from the data developers still lags behind the technical developments. This must be approached through understanding their perceptions as policy implementers, then addressed with support mechanisms and a consistent policy, which does not impose obstacles and recognises the obstacles and challenges data developers face.

That the creation of metadata is seen as a tedious and time-consuming exercise is one problem which must be overcome. Software engineering can offer limited solutions through metadata automation [68,69], but considerable work remains before this comes to fruition.

Even with metadata automation, and a complement of SDI services to support the delivery of metadata and spatial data, there still remain a number of concerns which must be addressed, including Intellectual Property Rights (IPR); residual licensed data rights for derived data; liability concerns; concerns over data quality (data creator and user); privacy and security issues; time and cost to anonymise data for release; time and cost for data delivery and for metadata record updates, especially descriptive level metadata; data transformation and harmonisation issues (scale, positional accuracy, projections, formats); legacy data concerns; properly dealing with VGI (Volunteered Geographic Information); issues related to performance, maintenance and enhancements of portals and repositories; data archiving requirements; data and software warehousing issues; long-term commitment and investment in the infrastructure; properly addressing revisions to standards; and solving any confusion about standards compliance and which standard to use.

There is no simple solution for confronting these issues. Intellectual Property Rights (IPR) is a primary concern especially amongst many in UK academia especially with regards to residual licensed data rights for derived data from the OS and other licensed data providers.

Liability and data quality concerns are shared between data developers and users. Good metadata records can address these, but liability concerns require legal consultation, especially for spatial data which could be used for emergency responses or monitoring the spread of infectious diseases. Disclaimers would be necessary, but with regards to the distribution of altered spatial data, will a data use disclaimer protect the original data provider? Will the data quality statement in the metadata record be accurate or updated correctly to reflect changes made to the spatial dataset? In academia, are researchers willing to expose their datasets to face further scrutiny? Anonymous sources have suggested that some data developers in academia might eschew metadata creation and data sharing due to concerns over the reliability of their data?

UK Health researchers face additional challenges with the creation of data which hold sensitive information and require anonymisation. The Data Protection Act 1998 [70] establishes legal requirements for health researchers to follow to ensure the confidentiality of personal information relating to research participants.

This reality imposes an expensive and time-consuming requirement on health researchers creating and using data which must be anonymised. There are also concerns about potential breaches (technological or legal) of data security and confidentiality [71].

The time and cost factors for anonymising, transforming and delivering data remains as a major obstacle for most data providers. These processes are often necessary to prepare data for deposit in a data repository. Updates made to resubmitted data must be recorded in accompanying metadata records.

Infrastructure and archiving are other concerns of data developers with regards to the reliability of SDI service providers. Can data developers be assured that their data can be properly managed, stored, displayed and disseminated? Failure of a service to make data accessible to users could lead to the data provider being directly contacted for data requests. Data developers may also use SDI services for the management of their data to save on infrastructure costs, hence an incentive for them to process and deliver data to an SDI service. However, data developers raise concerns about long-term data storage and migration of their data in the event the SDI service is discontinued.

Changes in data policies and revisions to standards also impose additional concerns on data developers. There are numerous standards, application profiles and competing interests across the various sectors. As noted, about 80 percent of all data have a spatial context [63]. Furthermore, privacy and security require considerable investment in the infrastructure that holds the data and metadata.

### Solutions

#### OSGeo

The OSGeo Geodata Committee [72] is playing an important role in providing open source solutions to address a number of the concerns and challenges data developers face. These proposed remedies fall under the auspices of the Public Geospatial Data Project [73], which offers the following in its mission statement:

• promote the use of open and free geospatial formats (GML, WMS, WFS-T) and metadata (Dublin Core, RDF, ISO 19115 through ISO 19139)

• promote public access to state-collected spatial data

• run an open spatial data repository with links to other open repositories

• present and explain licences for public spatial data through the collection of licences suitable for the publication of public spatial data.

The Open Knowledge Foundation [74] also endeavours to promote 'open knowledge' through its main principles of (i) free and open access to material; (ii) freedom to redistribute the material; (iii) freedom to reuse the material; and (iv) no legal, technological or social restrictions of the above based on who someone is or their field of endeavour (e.g. commercial or non-commercial).

The OSGeo Geodata Committee and Open Knowledge Foundation have taken important steps in the investigation and challenge of key issues which dissuade data sharing, especially with regards to various restrictions and licensing concerns. Another goal of the OSGeo Public Geospatial Project is to offer, in the future, a repository of reusable public geographic data that can support open source geospatial software projects [75]. These efforts can allow various GI-communities with limited resources to use the OSGeo resources and expertise to manage and disseminate their spatial data.

#### Go-Geo! Services

The Go-Geo! SDI service can provide UK academics and researchers with the resources to manage and disseminate their spatial data while addressing concerns about IPR, anonymised data and residual licensed data rights for derived data.

The UK AGMAP 2 metadata application profile can be extended to support the specific requirements of a discipline. As an example, UK AGMAP 2 could include elements which support the description of anonymised health data. A compliant application profile will include the ISO 19115's core mandatory elements, then be extended to include elements which address the needs of health researchers. It is at this level where consensus must be reached within the discipline to decide which elements should be created. Health portal metadata catalogues will be built to conform to this extended health data profile, but with the core ISO 19115 elements. This will ensure that searches conducted from other thematic portals will harvest discovery-level metadata from the health portals. These metadata records should contain information which directs users from other portals to the health portals where they can access the complete metadata records.

The Geodoc Metadata Editor tool allows users concerned about IPR and data privacy to store their metadata records in private directories, or publication on institutional nodes. The private Geodoc directory can also address the concerns and needs of medical researchers using and creating data with personal, sensitive information. Geodoc's export function also allows users to export metadata records to Dublin Core, DDI, UK GEMINI 2, FGDC and INSPIRE formats. As XML files, these records can uploaded with data to repositories, or shared between data users.

Derived data with residual licensing rights can be submitted to the closed version of ShareGeo which requires access via the Digimap Collections' authentication service. The ShareGeo Open version could also be extended to hold software source code and binaries, as well as software plug-ins, add-ons and extensions.

These Go-Geo! resources offer the UK academic community a no-cost and time effective solution for data management and sharing, because cost and time have been identified as primary reasons for not publishing metadata or sharing spatial data. Data developers are also invited to submit their spatial data to Go-Geo! services for extracting information to create partial metadata records, though this offer is seldom embraced.

### INSPIRE

The EC INSPIRE Directive [22] has a direct effect on the European GI-community as every Member State of the EU must transpose this Directive into law. The INSPIRE Regulations 2009 No 3157 came into force on 31 December 2009 and applies to England, Northern Ireland and Wales [76]; Scotland's Parliament enacted a complementary regulation which came into force on the same date [77].

The remit of the INSPIRE Directive is to "establish an infrastructure for spatial information in Europe to support Community environmental policies, and policies or activities which may have an impact on the environment." This, in effect, is a mandate for the creation of metadata and data sharing across the EU public sector in support of establishing an SDI for environmental information.

The environment has a direct impact on the daily lives of millions of EU citizens. Data modelling becomes an integral part of planning in the prevention and preparation of natural disasters which can pose serious risks to people, property and livelihoods. Experts building these models must be confident in the authenticity of the 'data' within the models, especially when situation awareness becomes critical and time is of the essence to. Inter-dependencies between environment, infrastructure and population create a situation where multi-criteria decisions become even more sensitive to accuracy of data and metadata.

Environment and health are linked with regards to direct effects of environmental disasters on people, and environmental data used for epidemiological research.

This reality would seem to provide the impetus for data developers across the EU to embrace metadata and data sharing especially those from the health community.

## Competing interests

The authors declare that they have no competing interests. TM works as the geospatial metadata co-ordinator for the Go-Geo! SDI Service.

## Authors' contributions

TM and MNKB conducted the literature review and wrote the manuscript. TM also provided detailed, firsthand information about the Go-Geo! SDI Service. Both authors read and approved the final version of the paper.
